# ERIC and WGS Typing of *Paenibacillus larvae* in Slovenia: Investigation of ERIC I Outbreaks

**DOI:** 10.3390/insects12040362

**Published:** 2021-04-19

**Authors:** Alenka Žugelj, Bojan Papić, Irena Zdovc, Urška Zajc, Majda Golob, Jana Avberšek, Darja Kušar

**Affiliations:** 1 Unit Maribor–Ptuj, Veterinary Faculty, National Veterinary Institute, University of Ljubljana, Šentiljska Cesta 109, SI-2000 Maribor, Slovenia; alenka.zugelj@vf.uni-lj.si; 2 Veterinary Faculty, Institute of Microbiology and Parasitology, University of Ljubljana, Gerbičeva 60, SI-1000 Ljubljana, Slovenia; bojan.papic@vf.uni-lj.si (B.P.); irena.zdovc@vf.uni-lj.si (I.Z.); urska.zajc@vf.uni-lj.si (U.Z.); majda.golob@vf.uni-lj.si (M.G.); jana.avbersek@vf.uni-lj.si (J.A.)

**Keywords:** *Paenibacillus larvae*, American foulbrood (AFB), ERIC-PCR, whole-genome sequencing (WGS), transmission, outbreak investigation

## Abstract

**Simple Summary:**

American foulbrood is a serious disease of honeybees caused by *Paenibacillus larvae*. ERIC-PCR is a widely used method for typing of *P. larvae* that currently divides it into five ERIC types (ERIC I–V); these differ in certain phenotypic characteristics—most importantly, virulence. In the first part of the study, we assessed the distribution of ERIC types in Slovenia in the period 2017–2019 on a set of 506 *P. larvae* isolates. We identified ERIC II as the predominant type (70.2%), followed by ERIC I (29.8%). In the second part of the study, we typed 59 outbreak-related ERIC I isolates using whole-genome sequencing, which revealed seven ERIC I-ST2 outbreak clusters (≤35 allele differences). The transmission of the outbreak clone within a 3-km radius was observed in all seven clusters and could be explained by the activity of honeybees. The transmission of the outbreak clone between geographically distant apiaries was observed in three clusters and could be explained by migratory beekeeping and trading of bee colonies. The present findings highlight the importance of beekeeping activities in the transmission of *P. larvae* over large geographic distances.

**Abstract:**

*Paenibacillus larvae* is the causative agent of American foulbrood (AFB), a fatal disease of honeybee brood. Here, we obtained 506 *P. larvae* isolates originating from honey or brood samples and from different geographic regions of Slovenia in the period 2017–2019. In the first part of the study, we conducted ERIC-PCR typing to assess the frequency of ERIC types in Slovenia. Capillary electrophoresis was used for the analysis of ERIC patterns, revealing good separation efficiency and enabling easy lane-to-lane comparisons. ERIC II was the predominant type (70.2%), followed by ERIC I (29.8%); two slightly altered ERIC I banding patterns were observed but were not considered relevant for the discrimination of ERIC types. No evident spatiotemporal clustering of ERIC types was observed. To assess the clonality of the outbreak-related *P. larvae* ERIC I isolates, 59 isolates of this type underwent whole-genome sequencing (WGS). Whole-genome multilocus sequence typing (wgMLST) revealed seven ERIC I-ST2 outbreak clusters (≤35 allele differences) with the median intra-outbreak diversity ranging from 7 to 27 allele differences. In all seven clusters, the transmission of *P. larvae* outbreak clone within a 3-km radius (AFB zone) was observed, which could be explained by the activity of honeybees. In three clusters, the transmission of the outbreak clone between geographically distant apiaries was revealed, which could be explained by the activities of beekeepers such as migratory beekeeping and trading of bee colonies. The present findings reinforce the importance of beekeeping activities in the transmission of *P. larvae*. WGS should be used as a reference typing method for the detection of *P. larvae* transmission clusters.

## 1. Introduction

American foulbrood (AFB) is a highly contagious and globally distributed disease affecting honeybee (*Apis mellifera*) broods. It is caused by a spore-forming bacterium *Paenibacillus larvae*. In Slovenia, as in many other countries, the detection, eradication, notification and prevention of AFB are regulated by the national legislation [1]. Under this legislation, all apiaries within a 3-km radius (AFB zone) from any AFB-positive apiary are subjected to clinical examination. A new AFB zone is denoted around each newly detected AFB-positive apiary, leading to the extension of the surveillance area. All AFB-positive honeybee colonies should be destroyed, leading to considerable economic losses to the beekeeping sector. Measures for the prevention of AFB are advised when spores are identified in honey or hive debris prior to the onset of clinical symptoms such as the shook swarm method and restriction of colony trade/movement, as well as thorough and consistent disinfection of the beekeeping equipment [2].

Genotyping methods are an integral part of the surveillance of infectious diseases, including AFB. Enterobacterial repetitive intergenic consensus (ERIC)-based PCR is a widely used method for typing of *P. larvae*, which classifies it into five ERIC types (I–V) [3,4]. These differ in biochemical, morphological and virulence characteristics [3,4,5,6]. ERIC types I and II are globally distributed and are epidemiologically the most important types [7,8,9].

Thus far, large-scale studies on the distribution of different ERIC types have been conducted in Austria [10], Belgium [11], Italy [12] and Japan [13]. In Italy, most AFB outbreaks were caused by ERIC I, but outbreaks caused by ERIC II or coinfections of ERIC I and II were also reported. In Austria, ERIC types I and II were represented in similar frequencies [10]. In Japan, an increase in the frequency of the ERIC II-ST10 type was observed in the last decade [13]. No data are currently available on the distribution and frequency of different ERIC types in Slovenia.

Due to the limited discriminatory power of ERIC-PCR, additional discrimination has been enabled by multilocus sequence typing (MLST) [7] and recently extended to core/whole-genome MLST (cg/wgMLST) [9,14]. Compared with ERIC-PCR and MLST, whole-genome sequencing (WGS) provides an insight into the complete genetic background of an organism, reaching an unprecedented discriminatory power [15]. Although WGS is increasingly employed in the surveillance and outbreak investigations of important human and zoonotic pathogens, it has been rarely used for the investigation of AFB outbreaks [9,14,16]. Thus, additional studies are needed to understand the intra- and inter-outbreak diversity of *P. larvae* isolates, as well as their ecology and transmission routes. 

The first aim of this study was to assess the frequency and spatiotemporal distribution of *P. larvae* ERIC types in Slovenia. Since several outbreak-related ERIC I isolates showed slightly altered banding patterns, the second aim was to assess the clonal relationship of ERIC I isolates using wgMLST.

## 2. Materials and Methods

### 2.1. Frequency and Spatiotemporal Distribution of ERIC Types

#### 2.1.1. Isolate Selection

To assess the frequency and spatiotemporal distribution of ERIC types, 506 *P. larvae* isolates originating from all geographic regions of Slovenia in the period 2017–2019 were selected for ERIC typing. The isolates were obtained during routine AFB surveillance and were part of *P. larvae* collection maintained by the Institute of Microbiology and Parasitology (Veterinary Faculty, National Veterinary Institute, Slovenia). They originated from honey (*n* = 203) or broods (*n* = 303) and were obtained from 263 apiaries, which were maintained by 232 beekeepers. For all isolates, metadata on the apiary, beekeeper and isolation date were collected (Table S1). The number of isolates per apiary ranged from 1 to 16; the number of ERIC-typed isolates per apiary was increased where we observed (i) a higher density of apiaries within the AFB zone or (ii) a slightly altered ERIC I banding pattern (Figure S1).

#### 2.1.2. Isolate Cultivation and DNA Extraction

The isolates were revived from frozen stocks (−70 °C) by streaking onto blood agar plates, followed by 72-h incubation at 37 °C. Species confirmation was performed by matrix-assisted laser desorption/ionization time-of-flight (MALDI-TOF) mass spectrometry (Microflex LT; Bruker Daltonics Inc., Leipzig, Germany). For ERIC-PCR and WGS, DNA was extracted using the DNeasy Blood & Tissue kit (Qiagen, Hilden, Germany) according to the protocol for Gram-positive bacteria.

#### 2.1.3. ERIC-PCR

ERIC typing was performed as previously described [3,17] with the slight modifications listed in reference [9]. Briefly, a 25-µL reaction was optimized to contain 1 × Multiplex PCR Master Mix (Qiagen, Hilden, Germany), 2 μM of each primer (ERIC1R and ERIC2) [18] and 5 μL of the DNA template. For the determination of ERIC patterns, amplicons were analyzed by the QIAxcel capillary electrophoresis (Qiagen, Hilden, Germany) using the QIAxcel DNA High-Resolution Kit, QX Alignment Marker 15–5000 bp, QX Size Marker 100 bp–2.5 kb, OM500 separation method and a sample injection time of 10 s. The prominent 2800-bp band present in ERIC II isolates but absent in ERIC I isolates [3,4] was considered relevant for the differentiation of ERIC types I and II. 

#### 2.1.4. Visualization of Spatiotemporal Data

The geographic location of each apiary was used to show the spatial distribution of the obtained isolates (from 1 to 16 isolates per apiary) and the isolation date to show their temporal distribution. The data were visualized using Microreact [19].

### 2.2. Phylogeny and Clonal Relationship of Outbreak-Related ERIC I Isolates

#### 2.2.1. Isolate Selection for WGS Typing

To assess the phylogeny and clonal relationship between the outbreak-related ERIC I isolates, including the isolates with slightly altered ERIC I banding patterns, 59/151 isolates of ERIC type I underwent WGS (Tables S1 and S2). The isolates originated from 29 apiaries with confirmed AFB, which were maintained by 27 beekeepers and were located in 13 different AFB zones covering several geographic regions of Slovenia during the period of 2017–2019 (Table S2). Additional epidemiological data relevant for the transmission of *P. larvae* due to the activities of beekeepers (i.e., beekeeper, migratory beekeeping and trading of bee colonies) were collected where available (Table S2). 

#### 2.2.2. WGS Typing

The extracted DNA was quantified by Qubit 3.0 (Thermo Fisher Scientific, Waltham, MA, USA) using the Qubit 1 × dsDNA HS assay kit. DNA libraries were prepared using the NEBNext Ultra DNA Sample Prep Master Mix kit (New England Biolabs, Ipswich, MA, USA). Paired-end (2 × 150 bp) sequencing was performed on the NovaSeq 6000 system (Illumina, San Diego, CA, USA) to a minimum coverage of 130×.

Raw reads were assembled using SPAdes version 3.7.1 implemented in BioNumerics version 7.6.3 (bioMérieux, Applied Maths NV, Sint-Martens-Latem, Belgium). The wgMLST quality assessment window was used to assess the quality of the obtained reads and assemblies; only the genomes passing the applied thresholds were included in the further analyses. The following thresholds were used: (i) the average read quality of *Q* > 30 and the expected coverage of >30 for the trimmed reads and (ii) *N*_50_ > 21,250 kb, number of contigs <380 and the total assembly length of ~4.1 Mb (range, 3.2–5.3 Mb) for the assemblies.

The wgMLST analysis was performed in BioNumerics by applying the stable wgMLST scheme for *P. larvae* consisting of 5745 loci [9]. Assembly-based and assembly-free allele calling were performed by applying the default parameters. The UPGMA tree and the minimum spanning tree (MST) based on the allele profiles of 5738-wgMLST loci (excluding the partial sequences of seven MLST loci) were constructed in BioNumerics with the allele calls considered as categorical values.

To assess the phylogeny and clonal relationship of the outbreak-related isolates with slightly altered ERIC I banding patterns (38/59 isolates; Table S2), a dataset of 179 publicly available *P. larvae* genomes (complete or draft) described in reference [9] was used and supplemented with WGS data of the 59 ERIC I isolates obtained in this study. The UPGMA wgMLST tree was constructed as described above and visualized and annotated using iTol version 4.4.2 [20]. 

#### 2.2.3. In Silico 7-Gene MLST

MLST sequence types (STs) were extracted from the assembled genomes of 59 ERIC I isolates using the MLST plugin implemented in BioNumerics; the *P. larvae* PubMLST nomenclature [21] based on seven previously described housekeeping genes [7] was used. 

## 3. Results

### 3.1. Frequency and Spatiotemporal Distribution of ERIC Types

**Figure 1:**
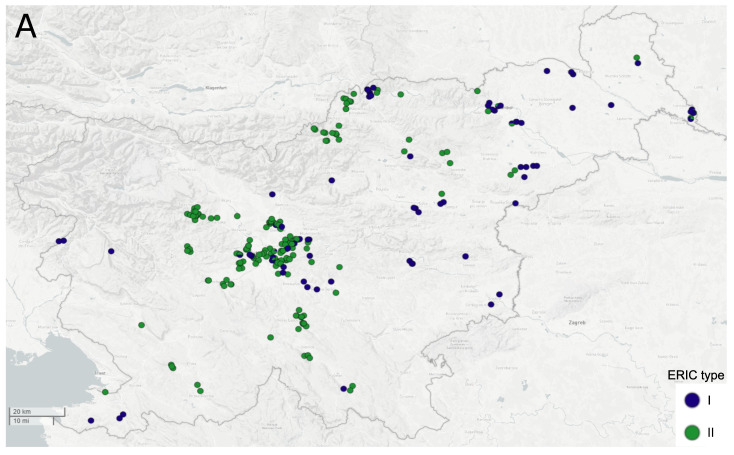

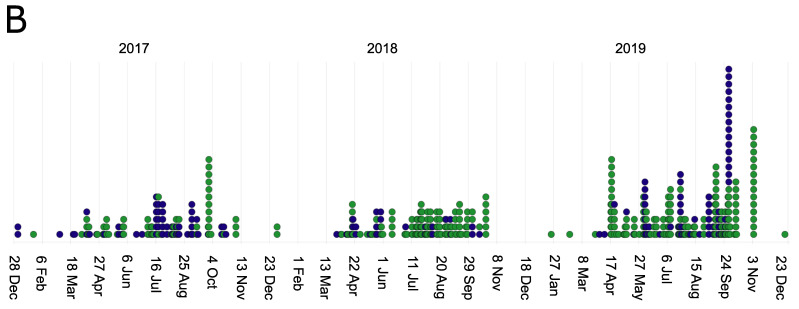
Spatial (**A**) and temporal (**B**) distribution of ERIC types in Slovenia. A total of 506 *Paenibacillus larvae* isolates from the 2017–2019 period were included into the analysis. Each circle represents an apiary (**A**) or isolate (**B**) and is colored according to ERIC type. Note that more than one isolate per apiary was obtained in 107 cases.

Among the studied 506 isolates, ERIC II was the predominant type (355/506; 70.2%), followed by ERIC I (151/506; 29.8%) (Table S1). In total, 74 ERIC I isolates exhibited a slightly altered ERIC I banding pattern; of these, 49 had an additional 600 bp and 25 an additional 650-bp band (Figure S1 and Table S1). The repeatability of these altered ERIC I banding patterns was confirmed by performing three independent ERIC-PCR replicates for one representative isolate per pattern. Figure 1 shows the spatiotemporal distribution of *P. larvae* ERIC types in Slovenia in 2017–2019. In general, both ERIC types were encountered in all geographic regions and over the entire study period. With regards to the sample type, ERIC II prevailed both in honey and brood samples (81.8% and 62.4%, respectively), followed by ERIC I (18.2% and 37.6%, respectively). In 107/263 apiaries where more than one isolate per apiary was typed, a mixed contamination or infection with ERIC types I and II was observed in 8/107 (7.5%) cases (Table S1). Since isolates from the same apiary represent possible epidemiological replicates, the prevalence of ERIC types in the 2017–2019 period was calculated on a set of 271 isolates originating from different apiaries and belonging to different ERIC types (Table S1). Table 1 shows the prevalence of ERIC I and ERIC II types in different years and further confirms the predominance of ERIC II type in all study years. 

### 3.2. Phylogeny and Clonal Relationship of Outbreak-Related ERIC I Isolates

**Figure 2:**
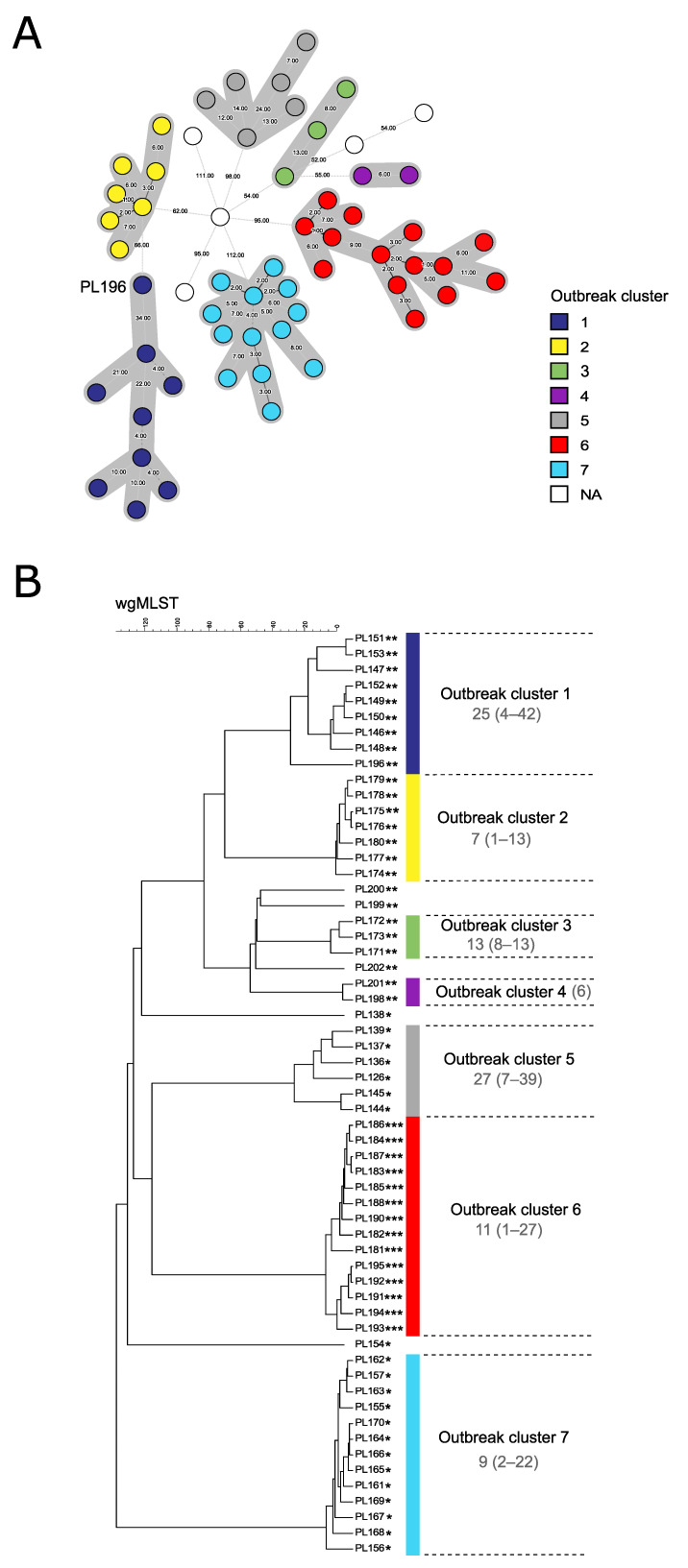
Whole-genome MLST analysis of 59 outbreak-related *Paenibacillus larvae* isolates of the ERIC I-ST2 type. Each isolate is colored according to the corresponding *P. larvae* outbreak cluster; the isolates without an assigned outbreak cluster are denoted “not assigned” (NA). The wgMLST analysis was based on 5738 wgMLST loci. (**A**) wgMLST minimum spanning tree. The connecting line lengths are proportional to the number of allele differences (AD) between isolates, which are denoted on the lines. Closely related isolates belonging to the same outbreak cluster (≤35 AD) are shaded in gray. Isolate PL196 is highlighted (for details, see Section 3.2.). (**B**) UPGMA wgMLST tree. Scale bar indicates the number of AD. The median pairwise AD between the isolates of the same outbreak cluster is shown on the right of the tree, with the minimum and maximum AD in parenthesis. *, isolates exhibiting a conventional ERIC I banding pattern; **, isolates exhibiting a slightly altered ERIC I pattern with an additional 600-bp band; ***, isolates exhibiting a slightly altered ERIC I pattern with an additional 650-bp band. For isolate metadata, see Table S2.

The MLST analysis of the 59 outbreak-related ERIC I isolates revealed that all of the investigated isolates belonged to ST2, including the isolates with altered the ERIC I banding patterns (38/59). The wgMLST analysis of the complete genome dataset (59 genomes of ERIC I type obtained in this study supplemented with 178 publicly available *P. larvae* genomes) revealed that all the investigated ERIC I genomes fell within the ERIC I clade and were clustered with the remaining ERIC I-ST2 genomes (Figure S2). The main clusters observed on the wgMLST tree of the 59 outbreak-related ERIC I isolates (Figure 2B) were generally congruent with the three observed ERIC I banding patterns (one conventional and two slightly altered), although they were not considered relevant, because they belonged to the same MLST type and clustered with the remaining ERIC I genomes in the wgMLST tree of the complete genome dataset (Figure S2). 

By applying the previously described single-linkage threshold of 35 allele differences (AD) on the wgMLST MST of *P. larvae* [9], seven outbreak clusters were identified (Figure 2A). Five isolates did not fall within any of the identified outbreak clusters (Figure 2). The median pairwise AD ranged from 7 (outbreak cluster 2) to 27 (outbreak cluster 5) (Figure 2A) and was comparable to that observed in the previously described ERIC I-ST2 outbreak (19 AD) [9]. The minimum and maximum pairwise AD between isolates per each cluster are shown in Figure 2B. The maximum pairwise genetic distance within an outbreak cluster was 42 AD (outbreak cluster 1). The minimum distance between the outbreak-related and nonrelated isolates on the wgMLST MST was 52 AD (Figure 2A). Of note, the clinical isolate PL196 belonging to the outbreak cluster 1 according to the applied threshold could not be linked to the remaining isolates of the respective outbreak cluster according to the available epidemiological data; thus, if this isolate is considered epidemiologically and genetically unrelated, this decreases the single-linkage threshold for cluster delineation to 24 AD and the minimum distance between outbreak-related and nonrelated isolates to 34 AD (Figure 2A).

### 3.3. P. larvae Transmission Routes

**Figure 3:**
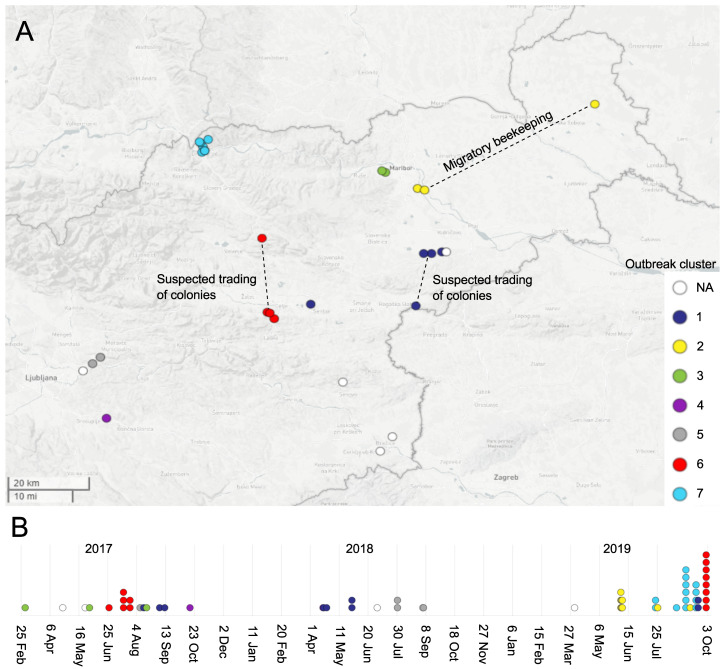
Map of the investigated AFB outbreaks caused by *Paenibacillus larvae* ERIC I-ST2 clones, Slovenia, 2017–2019. The figure shows spatial (**A**) and temporal (**B**) distributions of the 59 outbreak-related ERIC I isolates. Each circle represents an apiary (**A**) or isolate (**B**) and is colored according to the corresponding *P. larvae* outbreak cluster; the isolates without an assigned outbreak cluster are denoted “not assigned” (NA). Epidemiological data supporting the transmission of the outbreak clone over large geographic areas are highlighted (**A**). For isolate metadata, see Table S2.

In all the identified outbreak clusters, we observed the transmission of *P. larvae* outbreak clone between the apiaries that were located within the same AFB zone, which roughly corresponds to the honeybee flight distance and could thus be explained by the foraging activity of honeybees (Table S2). In addition, in outbreak clusters 1, 2 and 6, we observed the transmission of *P. larvae* outbreak clone over a large geographic area (i.e., between different AFB zones). For outbreak clone 2, such transmissions could be explained by migratory beekeeping (i.e., transfer of bees to a common foraging area), and for the outbreak clones 1 and 6, by the suspected (unregistered) trading of bee colonies (Table S2 and Figure 3). No intermixing of different outbreak clones was observed within a given AFB zone. Outbreak clusters 1 and 6 harbored isolates spanning the entire study period (2017–2019) and both sample types (honey and brood), and the remaining five outbreak clusters encompassed isolates from a single year (Figure 3). 

## 4. Discussion

The present study identified ERIC II (70.2%) as the predominant ERIC type in Slovenia in 2017–2019, regardless of the sample type, followed by ERIC I (29.8%). In addition, wgMLST revealed the existence of several ERIC I-ST2 outbreak clones that could not be distinguished using conventional typing methods (ERIC-PCR and MLST). The transmission between geographically distant (>3 km) apiaries was identified in three out of seven outbreak clones, highlighting the importance of beekeeping activities in the transmission of *P. larvae*.

### 4.1. Frequency and Spatiotemporal Distribution of ERIC Types 

Although ERIC II is considered to be less frequently encountered than ERIC I in Europe and worldwide [8,10,12], the present study identified ERIC II as the predominant ERIC type in Slovenia in all study years. The proportion of larvae developing into a ropy mass under cell cappings is higher for infections with ERIC I strains, which exhibit a slow-killing phenotype at the larval level. Contrary, ERIC II strains exhibit a fast-killing phenotype at the larval level; hence, false-negative diagnoses are likely to occur if AFB-diseased colonies are infected with ERIC II strains, because only a few infected cells may be present [3,5,6,22]. The high occurrence of ERIC II in Slovenia may thus result from a more successful identification and elimination of ERIC I-affected colonies as opposed to a more difficult identification of ERIC II-affected colonies, which serve as a better (and more often unrecognized) reservoir for the transmission of *P. larvae* spores within and between apiaries. 

In this study, the country-wide spatial distribution of ERIC types was considered rather than regional distribution for the following reasons. First, the national AFB surveillance system is based on a 3-km radius around the infected apiaries rather than any of the geographic classification systems. Second, the isolate panel was biased with regards to the geographic region, because it depended on the number of AFB reported cases and provided samples. Third, the transmission of *P. larvae* over large geographic regions was previously reported in Slovenia [9] and was also confirmed here, which can be explained by the activities of beekeepers (e.g., migratory beekeeping, which is common in Slovenia).

In this study, capillary electrophoresis was used for the ERIC-PCR amplicon analysis, which likely resulted in the better separation/visualization and comparability of ERIC patterns in comparison with conventional agarose gel electrophoresis used in previous studies [3,4,23,24]. The existence of two slightly altered ERIC I banding patterns was revealed in 38/59 WGS-typed outbreak-related ERIC I isolates; these were confirmed as ERIC I using MLST and WGS, since they clustered with the remaining ERIC I-ST2 isolates in the wgMLST tree (see Figures S1 and S2 and Table S2 for details). Thus, the less-conserved bands migrating at 600 or 650 bp observed in certain outbreak-related ERIC I isolates were not considered relevant for the discrimination of ERIC types. Only the prominent 2800-bp band present in ERIC II but absent in ERIC I isolates was used for the differentiation between ERIC types I and II, whereas the previously described 1200-bp faint band present in ERIC I but absent in ERIC II was not observed in this study. Taken together, these findings highlighted the importance of addressing only certain relevant bands when assigning the isolates to different ERIC types. The question of whether or not to consider novel bands in ERIC profiles as relevant points out the known limitations of ERIC-PCR regarding its difficult standardization and harmonization between different laboratories [8,25]. The description of novel ERIC types should be supported by genome-wide phylogeny and include in vitro pathogenicity studies, as exemplified by the recently described ERIC V type [4], which was also shown to form a separate clade on the MLST [4] and wgMLST [9] phylogenetic trees. In addition, we also observed here that the quality of the extracted DNA affects the obtained ERIC pattern, since the cell lysates of the *P. larvae* isolates proved unsuitable for ERIC-PCR (data not shown).

Since all the outbreak-related isolates were of the ERIC I-ST2 type, this study confirmed the limited discriminatory power of ERIC-PCR and MLST, both of which failed to discriminate between the identified outbreak clones. Although MLST was shown to provide a higher discriminatory power compared with ERIC-PCR [7], it could not differentiate between the identified outbreak clusters. Due to its unprecedented discriminatory power, WGS should be used as a reference method to detect *P. larvae* transmission clusters, as already shown in previous studies [9,16]. 

### 4.2. Phylogeny, Clonal Relationship and Transmission of Outbreak-Related ERIC I Isolates

The wgMLST analysis enabled a clear delineation of seven ERIC I-ST2 outbreak clusters. The median intra-outbreak diversity of *P. larvae* observed in this study was comparable to that reported in previous studies [9,14]. The present study reinforces the previously proposed 35-allele threshold (as determined by wgMLST) to delineate the outbreak clusters [9] but should allow for some flexibility due to technical and biological variations, as extensively discussed elsewhere [9,14,26,27]. In the present study, BioNumerics was used in combination with a stable wgMLST scheme for *P. larvae* [9], facilitating the harmonization and standardization of WGS typing tools and data interpretation in order to improve the surveillance of this important honeybee pathogen. The identified wgMLST clusters can be further investigated by a whole-genome single-nucleotide polymorphism (wgSNP) analysis, which may provide additional confirmation of the identified clusters. We previously showed that allele-based and wgSNP-based approaches both gave congruent results with regards to the outbreak cluster delineation of *P. larvae* isolates [9].

In outbreak clusters 1 and 6, the outbreak clone was observed throughout the entire study period (2017–2019), suggesting its persistence within the AFB zone(s). This could be explained by the high resilience of *P. larvae* spores and insufficient/inadequate preventive measures [8]. Moreover, three out of seven outbreak clones were not limited to a single AFB zone, suggesting the transmission of *P. larvae* outbreak clones over larger geographic distances. The latter most likely results from beekeeping activities, which may remain overlooked under the current national legislation that focuses on the examination of apiaries within the AFB zone. Taken together, the present findings call for the implementation of an improved country-wide surveillance protocol for the early diagnosis of AFB (e.g., monitoring of honey and hive debris samples) to detect *P. larvae*-positive apiaries without clinical symptoms of AFB, followed by rigorous prevention and eradication measures in these apiaries, which could decrease the frequency of such recurrent outbreaks. In this manner, apiaries with an increased risk of developing AFB would be identified regardless of their location from the current AFB zone(s). 

Genotyping results should be interpreted in combination with epidemiological data for the final confirmation of outbreaks and transmission events. In this study, the transmission of *P. larvae* outbreak clones within the AFB zone could be explained by the activity of honeybees (e.g., foraging, robbing and drifting). The transmission between geographically distant (>3 km) apiaries, which was observed in three outbreak clones, could be explained by migratory beekeeping (transfer of honeybee colonies to a common foraging area) or suspected trading of colonies that was not reported to the authority. In Slovenia, all migratory beekeeping activities should be reported to the national authority; nonetheless, the possibility of illegal (unreported) transfer of bee colonies cannot be ruled out. The present findings restate the importance of collecting extensive epidemiological data on the adopted beekeeping technologies and practices, as well as the participation of beekeepers themselves, to report migratory beekeeping activities and clinical symptoms of AFB in order to control the disease.

## 5. Conclusions

ERIC II was recognized as the predominant ERIC type in Slovenia. WGS typing enabled a clear delineation of the ERIC I-ST2 outbreak clusters. In addition to the transmission of *P. larvae* outbreak clone within the AFB zone, several cases of transmission between geographically distant apiaries were revealed, indicating the role of beekeeping activities in the transmission of *P. larvae*. The present study improves our understanding of the genetic diversity and transmission dynamics of *P. larvae,* facilitating future surveillance of this important bee pathogen. WGS data should be interpreted in combination with epidemiological data for final confirmation of transmission events. 

## Figures and Tables

**Table 1:** Prevalence of the ERIC types over time. The prevalence was calculated on a set of 271 *Paenibacillus larvae* isolates originating from different apiaries and belonging to different ERIC types (for details, see Table S1).

Year	ERIC I (%)	ERIC II (%)	Total No. of Isolates
2017	44.2	55.8	86
2018	14.5	85.5	83
2019	26.5	73.5	102

## Data Availability

All WGS data obtained in this study have been deposited in the NCBI Sequence Read Archive (SRA) database under BioProject accession number PRJNA648798.

## Supplementary Materials

The following are available online at https://www.mdpi.com/article/10.3390/insects12040362/s1: Figure S1. ERIC patterns of the analyzed *Paenibacillus larvae* isolates obtained by QIAxcel (Qiagen, Hilden, Germany) capillary electrophoresis. ERIC types are denoted above the patterns. Green arrows denote the bands that were considered important for the differentiation of ERIC types, whereas red arrows denote the bands that occurred in certain outbreak-related ERIC I isolates but were not considered important for the differentiation of ERIC types [3,4]. Columns 1 and 2: *P. larvae* outbreak-related field strains exhibiting a slightly altered ERIC I banding pattern. Columns 3–7 (Control strains): *P. larvae* strains ATCC 9545 (ERIC I), CCUG 49660A (ERIC II), LMG 16252 (ERIC III), LMG 15974 (ERIC IV) and DSM 106052 (ERIC V). Column 8 (M): QX DNA Size Marker 100 bp–2.5 kb (Qiagen, Hilden, Germany). See Table S1, column “Comment (ERIC pattern)”, for details. Figure S2. Whole-genome phylogenetic tree of 238 *Paenibacillus larvae* genomes. The UPGMA wgMLST tree was constructed based on 5738 loci and is annotated with ERIC and MLST types. The genomes are colored according to ERIC type. The 59 outbreak-related ERIC I isolates analyzed in this study are denoted by red stars. For genome metadata, see Table S2. Table S1. Metadata of the 506 *Paenibacillus larvae* isolates from Slovenia used for assessing the frequency and spatiotemporal distribution of ERIC types. Table S2. Metadata of the 59 outbreak-related *Paenibacillus larvae* isolates of ERIC I-ST2 type analyzed in this study. NA, not applicable; ND, no data.
