# Supplementary figures and images for: ERIC and WGS Typing of Paenibacillus larvae in Slovenia: Investigation of ERIC I Outbreaks
Source: Insects. 2021 Apr 19;12(4):362. doi: 10.3390/insects12040362 (PMC8072612; doi:10.3390/insects12040362)

| Field strains |   | Control strains |    |     |    |   |   |
|---------------|---|-----------------|----|-----|----|---|---|
| I             | I | I               | II | III | IV | V | M |

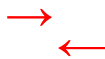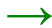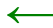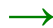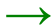

Supplement: Supplementary file 1 [file insects-12-00362-s001.zip › Figure_S1.pdf]

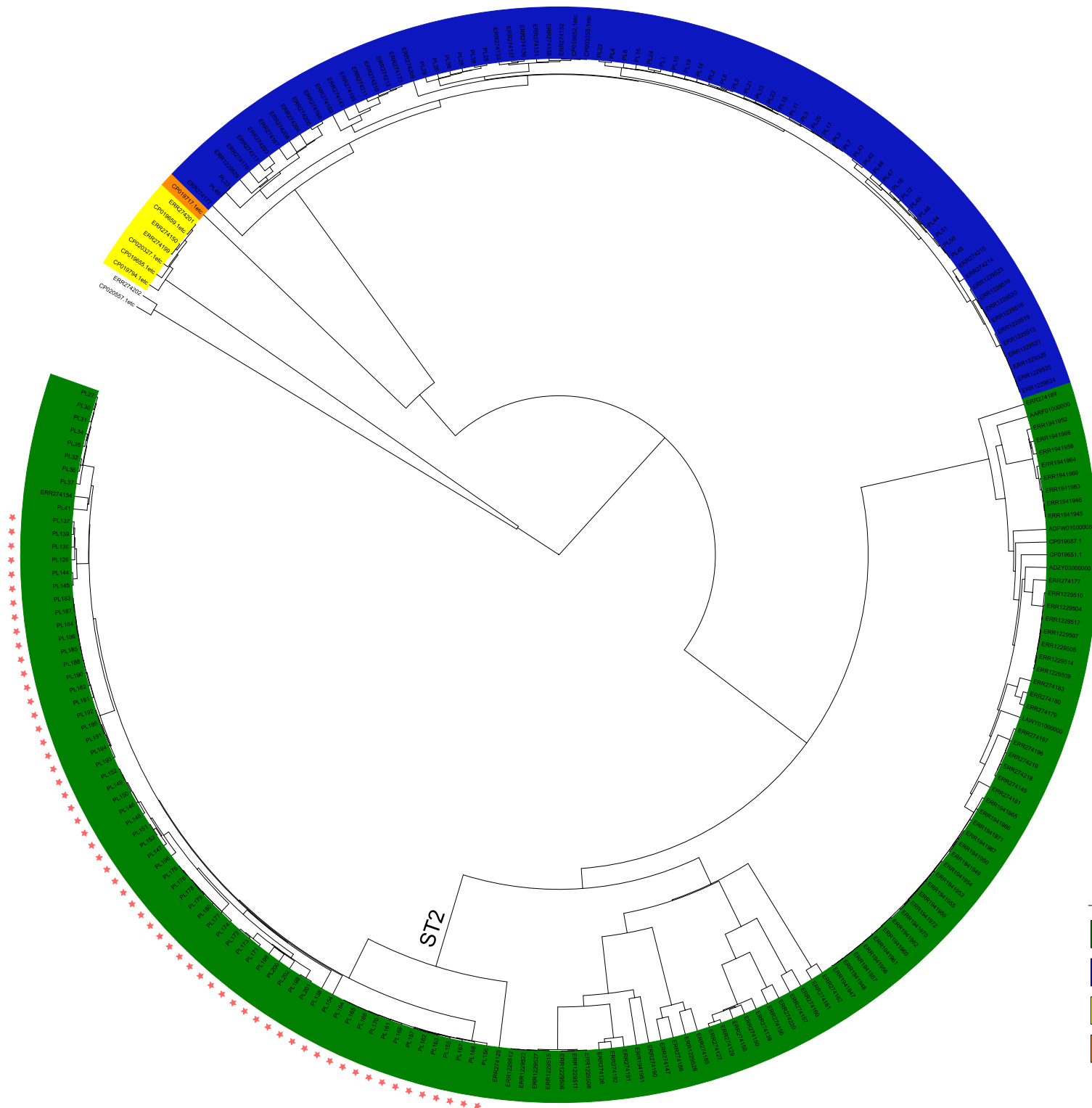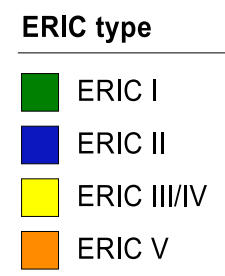

Supplement: Supplementary file 1 [file insects-12-00362-s001.zip › Figure_S2.pdf]
